# Lemierre Syndrome Complicated by Emphysematous Osteomyelitis

**DOI:** 10.7759/cureus.43719

**Published:** 2023-08-18

**Authors:** Jesse J Cotton, Frederick M Lang, Chinelo Onyilofor, Abigail Ritter, Shauna Gunaratne

**Affiliations:** 1 Infectious Disease, Columbia University College of Physicians and Surgeons, New York, USA; 2 Internal Medicine, Columbia Irving Medical Center, New York, USA; 3 Infectious Disease, Columbia Irving Medical Center, New York, USA

**Keywords:** septic pulmonary emboli, septic emboli, antibiotics, emphysematous osteomyelitis, atypical lemierre syndrome

## Abstract

Lemierre syndrome is characterized by severe pharyngitis, internal jugular vein thrombosis, and septic emboli. We present a case of emphysematous osteomyelitis secondary to Lemierre syndrome in a 27-year-old previously healthy man. Despite the high mortality associated with these conditions, full symptom resolution can be achieved with early diagnosis and aggressive management.

## Introduction

Lemierre syndrome (LS) is characterized by severe pharyngitis, septic thrombophlebitis of the internal jugular vein (IJV), and multi-organ sequelae from septic emboli and metastatic infection. The condition is most commonly caused by *Fusobacterium necrophorum* and usually occurs in young, healthy individuals [1]. Now much rarer after the introduction of antibiotics in the 1940s, the annual incidence is currently 0.8-3.6 cases per million. Fewer than 750 cases have been described in the literature since 2000, leading to a paucity in our understanding of the syndrome’s pathogenesis and possible complications [2]. Here, we describe a young man who was diagnosed with LS and demonstrated intraosseous gas within the manubrium indicative of emphysematous osteomyelitis (EO), an extremely rare finding with fewer than 75 cases reported in the literature [3].

## Case presentation

A 27-year-old man with no significant medical history was in his usual state of health when he developed a fever and sinus congestion (day zero). He denied sick contacts, recent travel, or intravenous drug use. He visited a local walk-in clinic and tested negative for SARS-CoV-2, influenza, and Group A *Streptococcus*. He then developed diffuse myalgias, a headache, productive cough, night sweats, and chest and throat pain. By day four, he was experiencing severe dyspnea and had trouble speaking and swallowing, prompting a presentation at a local hospital.

**Figure 1:**
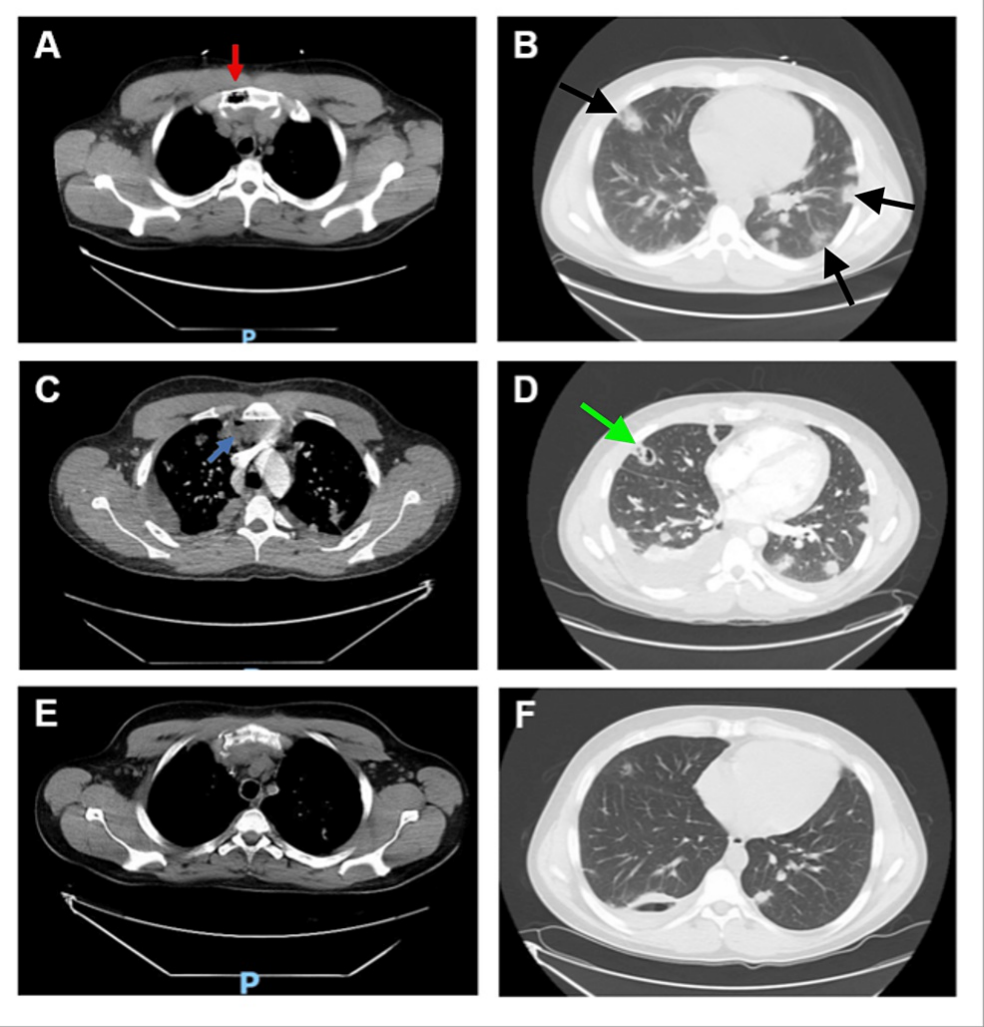
Computed tomography (CT) scans of the chest on day Four (A, default window; B, lung window), day 11 (C, default window; D, lung window), and day 53 (E, default window; F, lung window). On day four, the CT chest demonstrated intraosseous gas and edema in the right aspect of the manubrium of the sternum (red arrow), consistent with emphysematous osteomyelitis (A). Widespread nodules were visible in the bilateral lung fields, consistent with septic emboli (black arrows) (B). On day 11, CT chest demonstrated new mediastinal abscesses in front of and behind the sternum (blue arrow) (C) and new right and left pleural effusions (C, D). Some pulmonary nodules had evolved into cavitary lesions (green arrow) (D). On day 53, the air within the manubrium had resolved (E), and marked improvement in the lung lesions was observed (F).

In the emergency department, he demonstrated hypotension (84/57 mmHg), tachycardia (122 bpm), fever of 40.8°C, and SpO2 95% on a 2L nasal cannula. The exam revealed an ill-appearing, diaphoretic man with an erythematous posterior oropharynx, dry mucous membranes, and tenderness over the sternum. Laboratory tests showed leukocytosis (16x103 cells/μL), profound thrombocytopenia (5x103 cells/μL), severe hyponatremia (118 mmol/L), and acute kidney injury (AKI) (creatinine of 3.2 mg/dL). He was admitted to the intensive care unit (ICU) for septic shock and required up to 8L supplemental oxygen by a face mask at 35% FiO2 during the following days. Computed tomography (CT) imaging showed intraosseous gas within the manubrium consistent with emphysematous osteomyelitis (Figure 1A), innumerable bilateral nodules throughout the lungs indicative of septic emboli (Figure 1B), and hepatosplenomegaly (liver of 20 cm, spleen of 15 cm). Magnetic resonance imaging confirmed intraosseous gas within the manubrium. Blood cultures grew *F. necrophorum* and *S. anginosu*s, and throat cultures grew *S. anginosus*. Other diagnostic studies, including CT head, transthoracic echocardiography (TTE), and bilateral IJV ultrasound, were unrevealing. The patient was treated with vancomycin, piperacillin-tazobactam, platelet transfusions, intravenous fluids, and electrolyte repletion, leading to the resolution of hyponatremia and AKI. He was transferred to our institution on day seven for further management.

On arrival at our ICU, he was afebrile and hemodynamically stable, with SpO2 at 94% on a 2L nasal cannula. He had dyspnea while speaking, but his throat and chest pain had improved. Platelets remained low (16x103 cells/μL). Our infectious disease team was consulted and felt the presentation was consistent with LS. Bilateral IJV ultrasound was repeated but negative for thrombosis. TTE showed a reduced ejection fraction of 40%. Blood culture sensitivities from the outside hospital showed a *S. anginosus* isolate susceptible to penicillin (minimum inhibitory concentration 0.012 μg/mL) and an *F. necrophorum* isolate that was beta-lactamase negative, so the patient's treatment was narrowed to ampicillin-sulbactam.

He was transferred to the floor on day eight. In the subsequent days, his thrombocytopenia resolved. However, fevers persisted (Tmax 39.3°C), and he complained of sternal pain, a productive cough, and orthopnea, prompting the broadening of antibiotics to vancomycin and piperacillin-tazobactam. CT chest on day 11 revealed a 6 cm retrosternal abscess, a 4.6 cm pre-sternal abscess, and moderate right and small left pleural effusions (Figure 1C). Pulmonary septic emboli and intraosseous air with lytic bone destruction in the sternum were redemonstrated (Figures 1C-1D). Given these findings, intravenous metronidazole was added for further anaerobic coverage. On day 12, the pulmonology team performed a bedside right-sided thoracocentesis, draining 18cc of serosanguinous fluid. The thoracic surgery team then performed an abscess washout in the operating room, with the placement of three Jackson-Pratt drains. To relieve the patient’s clot burden, therapeutic-level enoxaparin was administered.

Over the following days, the patient was able to ambulate, but fevers persisted. Repeat blood cultures remained negative. CT chest on day 16 revealed a right-sided hydropneumothorax and expanded right upper lobe opacities. A right-sided chest tube was placed for drainage. Anticoagulation was held. On day 20, the patient required the placement of a second chest tube for further right-sided effusion drainage. On day 21, he developed a faint morbilliform rash on his trunk and arms, without eosinophilia, systemic syndrome, or mucosal involvement. Dermatology was consulted and felt these findings were consistent with a drug eruption; antibiotics were switched to intravenous ertapenem. Over the following days, tPA was administered to the chest tubes to enhance drainage, and his fevers finally resolved. By day 26, all drains and tubes had been removed. TTE demonstrated a recovered ejection fraction (50-55%). The patient was discharged on day 29 with a PICC line for at-home antibiotic administration.

Outpatient CT chest on day 53 demonstrated the resolution of air in the sternum, markedly reduced lung nodules, and no major pleural effusions (Figures 1E-1F). At the outpatient follow-up on day 84, the patient was asymptomatic and had returned to work. The PICC line was removed, and antibiotics were discontinued.

## Discussion

This report describes an unusual case of Lemierre syndrome complicated by sternal emphysematous osteomyelitis and mediastinal abscesses. Organisms isolated from the blood were *F. necrophorum* and *S. anginosus*, a viridans subgroup streptococcus previously associated with LS [4]. The typical history of high fever and severe pharyngitis was present, and, although no IJV thrombus was identified, indirect evidence of such thrombus was observed as septic emboli throughout the lungs. Profound thrombocytopenia and hyponatremia, as well as a reduced ejection fraction, were observed and were likely nonspecific signs of sepsis.

LS was first described in 1936 by Andrew Lemierre, a professor of bacteriology from Paris who reported a series of 20 patients [5]. While the mortality rate observed then was ~90%, mortality has been 2%-18% in the post-antibiotic era. Although the lungs are primarily affected, widespread manifestations of septic emboli have been described, including septic arthritis, intracranial thrombosis, and infective endocarditis [2,6]. To our knowledge, this is the third report of emphysematous osteomyelitis associated with LS. One case was located in the T12 vertebral body and was caused by *Fusobacterium nucleatum* in a 20-year-old woman who, similar to our patient, had no significant medical history and presented without IJV thrombophlebitis [7]. The other case was caused by *F. necrophorum* in a 20-year-old woman. EO was identified in the clavicle, and, similar to our case, *Cutibacterium acnes* grew from a bone swab obtained during surgical debridement [8].

First described in 1981, emphysematous osteomyelitis is extremely rare and associated with poor outcomes [8,9]. In the largest review to date of 49 cases, only one (2%) infection occurred in the sternum, and seven (14%) cases had no predisposing factors. Thirty-one (63%) cases required surgical intervention, and the mortality rate was 34%. While no cases involved *S. anginosus*, eight (16%) involved *Streptococcus *spp., seven of which were part of a polymicrobial infection. One (2%) case involved polymicrobial infection including Cutibacterium spp. Four (8%) cases involved monomicrobial infection with *F. necrophorum*, a known producer of hydrogen sulfide gas [3,8]. Based on these data more directly implicating *F. necrophorum* as a culprit organism compared with other microbes, we hypothesize that *F. necrophorum *was the driver of EO in our patient. It is likely that our patient’s bone infection occurred via hematogenous spread rather than via contiguous spread from cervical tissue, given the lack of deep neck infection on initial imaging. Interestingly, *C. acnes* was isolated from the mediastinal abscesses, rather than *F. necrophorum* or *S. anginosus*. *C. acnes* is an anaerobic commensal bacterium that colonizes hair follicles and causes acne vulgaris and prosthetic material infections [10]. Because EO was observed before abscess formation, it is likely that the initial metastatic infection to the bone by *F. necrophorum* created an optimal environment for secondary infiltration by *C. acnes* into the sternal space. We suspect a similar mechanism occurred in the previous case of LS-associated EO where *C. acnes* was isolated [8].

Regarding the management of this case, broad-spectrum intravenous antibiotics, electrolyte repletion, and fluid resuscitation were key for initial stabilization. However, symptoms persisted until surgical washout and percutaneous drainages were performed, demonstrating the importance of repeating imaging and obtaining source control. Additionally, anticoagulation has been used previously in LS, with minimal risk of major bleeding demonstrated [2,6,11,12]. We initially elected to administer anticoagulation to improve pulmonary clot burden; however, we withdrew this treatment given the lack of an IJV thrombus and the unclear benefit demonstrated in the literature [2,6,11,12]. Ultimately, no further thrombotic events were observed, and lung lesions improved without anticoagulation.

## Conclusions

We demonstrate that the Lemierre syndrome can be complicated by emphysematous osteomyelitis in a previously healthy young patient, likely via the hematogenous spread of the pathogen. Appropriate antibiotic choice, repeated procedures to achieve source control, and evaluation of anticoagulation were key in the management of this complex case. Despite high mortality rates typically associated with LS and emphysematous osteomyelitis, excellent outcomes with full symptom resolution can be achieved with early recognition of these conditions and appropriate antibiotic and surgical management.

## References

[1] Carius BM, Koyfman A, Long B (2022). High risk and low prevalence diseases: Lemierre's syndrome. Am J Emerg Med.

[2] Pleming W, Barco S, Voci D (2022). Cardiac and cerebral arterial complications of Lemierre syndrome: results from a systematic review and individual patient data meta-analysis. Hamostaseologie.

[3] Sulyma V, Sribniak A, Bihun R, Sribniak Z (2020). Emphysematous osteomyelitis: review of the literature. Ortop Traumatol Rehabil.

[4] Santos FV, Pires SX, Pereira C, Gonçalves L, Martins S, Aragão I (2020). Deep neck space infection and Lemierre's syndrome caused by Streptococcus anginosus: a case report. IDCases.

[5] Lemierre A (1936). On certain septicæmias due to anaerobic organisms. Lancet.

[6] Valerio L, Zane F, Sacco C (2021). Patients with Lemierre syndrome have a high risk of new thromboembolic complications, clinical sequelae and death: an analysis of 712 cases. J Intern Med.

[7] Nguyen HD, Whitley-Williams PN, Uppaluri LP, Sangani J, Simon ML, Baig AS (2022). Case report of atypical Lemierre's syndrome associated with Fusobacterium nucleatum infection without internal or external jugular venous thrombophlebitis. Respir Med Case Rep.

[8] Litt MJ, Gaffney R, Vaidya A, Montgomery MW (2021). Hard to swallow. N Engl J Med.

[9] Ono R, Uehara K, Kitagawa I (2018). Emphysematous osteomyelitis of the spine: a case report and literature review. Intern Med.

[10] Mayslich C, Grange PA, Dupin N (2021). Cutibacterium acnes as an opportunistic pathogen: an update of its virulence-associated factors. Microorganisms.

[11] Phua CK, Chadachan VM, Acharya R (2013). Lemierre syndrome-should we anticoagulate? A case report and review of the literature. Int J Angiol.

[12] Nygren D, Elf J, Torisson G, Holm K (2021). Jugular vein thrombosis and anticoagulation therapy in Lemierre's syndrome—a post hoc observational and population-based study of 82 patients. Open Forum Infect Dis.

